# Exposure to Commonly Used Drugs and the Risk of Gastric Cancer: An Umbrella Review of Meta-Analyses

**DOI:** 10.3390/cancers15020372

**Published:** 2023-01-06

**Authors:** Xiao Bai, Si-Qi Ding, Xue-Ping Zhang, Ming-Hao Han, Dong-Qiu Dai

**Affiliations:** 1 Department of Gastrointestinal Surgery, The Fourth Affiliated Hospital of China Medical University, Shenyang 110032, China; 2 Cancer Center, The Fourth Affiliated Hospital of China Medical University, Shenyang 110032, China

**Keywords:** drug, gastric cancer, risk, umbrella review

## Abstract

**Simple Summary:**

To date, several systematic reviews and meta-analyses have explored associations between commonly used drugs and gastric cancer (GC) risk, with inconsistent conclusions on relationships and methodological quality. No attempts have been made to quantify the credibility of these findings. Hence, it is necessary to compare the results of individual reviews by looking into existing systematic reviews and meta-analyses, providing an overview of the findings of a particular association. This is the first umbrella review to evaluate the validity and credibility of evidence from previously published systematic reviews and meta-analyses on observational studies and to identify associations between commonly used drugs and GC risk and its subtypes.

**Abstract:**

Recently, attention has been paid to some medications and gastric cancer (GC) risk. This review aimed to evaluate associations between commonly used drugs and GC risk and to grade evidence from published systematic reviews and meta-analyses. This umbrella review was registered in PROSPERO (CRD42022320276). The systematic reviews and meta-analyses of observational studies were retrieved by searching Embase, PubMed, and Web of Science. The evidence strength of commonly used drugs and GC risk was categorized into four grades: weak, suggestive, highly suggestive, and strong. Of 19 associations between commonly used drugs and GC risk and its subtypes, none was supported by convincing or highly suggestive evidence. The risk of GC related to non-steroidal anti-inflammatory drugs (NSAIDs), non-aspirin NSAIDs, and acid-suppressive drugs, as well as the risk of non-cardia GC related to NSAIDs and aspirin, was supported by suggestive evidence. The results showed that a reduced GC risk was associated with two drug types (NSAIDs and non-aspirin NSAIDs), and an increased GC risk was associated with acid-suppressing drugs at the suggestive evidence level. Moreover, NSAIDs and aspirin reduced non-cardia GC risk as supported by suggestive evidence. However, the evidence supporting statins or metformin in reducing GC risk was weak, and thus future studies are required to clarify these associations.

## 1. Introduction

Globally, it was estimated that over one million new incidences of gastric cancer (GC) and approximately 769,000 deaths were reported in 2020, and GC ranked as the fourth-highest mortality rate and the fifth-highest incidence [1]. According to the International Agency for Research on Cancer Incidence, Eastern Europe and Eastern Asia (China and Japan) have the highest rates. According to 2030 predictions, the GC incidence rate will decrease in most countries [2], however, GC is still a major global cancer burden at present [3]. With improved screening programs and advances in both surgical and endoscopic techniques, the 5-year survival rate for early GC tends to reach >90% [4]. Current treatment strategies include surgery, chemoradiotherapy, molecular-targeted therapy, and immunotherapy [5,6]. Unfortunately, GC is usually diagnosed at advanced stages, often with a poor prognosis. Similarly, the prognosis for patients with recurrent or metastatic disease is poor, with a median survival of eight months [7]. Thus, preventative measures are crucial for controlling disease incidence.

The eradication of *Helicobacter pylori* (*H. pylori*) is a pivotal therapeutic step against GC [8]. The established risk factors for GC, apart from *H. pylori* infection, include family history, salted food, alcohol, and smoking [9,10,11,12]. Recently, several studies reported that some commonly used drugs had potential links with GC risk, including cardiovascular medications, antidiabetics, and acid suppressants [13,14,15]. However, the preventative effects of medications such as statins, aspirin, metformin, and proton pump inhibitors (PPIs) against GC remain controversial. Additionally, several systematic reviews and meta-analyses investigated medication use and GC risk [16,17], but in fact, no attempts have been made to quantify the credibility of these findings. Given the uncertainty of observational research, this quantification of evidence is both timely and critical [18,19]. An umbrella review can be used to summarize evidence from numerous, same-topic meta-analyses, and rank the evidence of each association [20,21]; therefore, such an umbrella review was conducted to generate a better understanding of the evidence’s strength per association.

Overall, this review evaluated the validity and credibility of evidence from previously published systematic reviews and meta-analyses on observational studies and identified associations between commonly used drugs and GC risk.

## 2. Materials and Methods

This umbrella review is registered at PROSPERO (CRD42022320276). It was conducted following guidelines from the Preferred Reporting Items of Systematic Reviews and Meta-Analyses [22].

### 2.1. Search Strategy and Selection Criteria

Web of Science, Embase, and PubMed (last updated on 14 January 2022) were searched to retrieve all systematic reviews and meta-analyses of observational studies focusing on associations between commonly used drugs and GC risk (Table S1). All references in identified studies were manually searched to identify eligible articles.

Two investigators (X.B. and S.-Q.D.) independently screened titles and abstracts, and checked full texts. All disagreements were figured out by three authors (X.B., S.-Q.D. and D.-Q.D.) through discussion. Eligibility criteria included: (1) systematic reviews and meta-analyses of observational studies measuring associations between commonly used drugs and GC incidence in any population; (2) results from subgroup analyses stratified by drug type; (3) studies focusing on GC subtypes.

Exclusion criteria included: (1) articles that did not examine the use of any commonly drugs; (2) articles that did not assess outcomes of interest (including but not limited to progression-free survival, disease-free survival, overall survival, and mortality of GC); (3) articles that described some unconventional drugs (such as chemotherapeutic drug, targeted drug, and traditional Chinese medicine); (4) meta-analyses including less than three original studies. In addition, non-English articles, animal studies, and genetic studies were excluded as well. If the same association was examined by two or more meta-analyses, associations in different meta-analyses were considered to be duplicate associations; therefore, the meta-analysis with the largest sample size was selected to avoid overlap, as previously indicated [23,24,25].

### 2.2. Data Extraction

Two authors (X.B. and S.-Q.D.) carried out extraction independently, and three authors (X.B., S.-Q.D. and D.-Q.D.) resolved discrepancies by consensus. The first author’s name, year of publication, country, drug type, outcome (GC or its subtypes), comparison, total participants, number of GC cases, and number of included studies were extracted from each eligible meta-analysis. The first author’s name, year of publication, number of subjects and cases, as well as maximally adjusted effect size, including corresponding 95% confidence interval (CI), hazard ratio, odds ratio, and relative risk, were recorded from each primary study included in the meta-analysis for further analysis.

### 2.3. Quality Assessment

A Measurement Tool to Assess Systematic Reviews (AMSTAR) version 2.0, which contained 16 items, was used by two researchers (X.B. and S.-Q.D.) to evaluate the study quality [26]. Disagreements were resolved by discussion. All 16 items used to conduct systematic reviews and meta-analyses were important, but seven were critical for reviewing validity and conclusions—also known as critical domains. On this basis, AMSTAR-2 was used to define the quality of a systematic review, being critically low (more than one critical defect with or without non-critical weaknesses), low (one critical defect with or without non-critical weaknesses), moderate (more than one non-critical weakness), or high (no or one non-critical weakness).

### 2.4. Statistical Analysis

First, the effect size of each study included in each meta-analysis was extracted, and a random-effect model was used to assess the effect size with 95% CI for each association. The corresponding *p*-values for summary effects were calculated at the same time [27]. Heterogeneity was evaluated by I^2^ statistics; an I^2^ > 50% value indicated significant heterogeneity and an I^2^ > 75% indicated high heterogeneity [28,29]. To be more conservative, a 95% prediction interval (PI) was calculated to analyze inter-study heterogeneity and to assess the uncertainty of summary effect sizes in the random-effect model. The expected effect size range of future studies was also predicted by 95% PI [30].

Then, Egger’s regression asymmetry tests were used to identify small-study effects, reflecting heterogeneity, genuine opportunity, or other reasons for differences between small and large studies [31,32,33]. A small-study effect bias was indicated by *p*-value for Egger’s test < 0.10.

Excessive significance bias was applied after accounting for publication bias, selective reporting bias and other potential biases. Whether the expected number (E) with significant results was less than the observed number (O) with significant results was compared [31]. A non-central t distribution was used to calculate an E value from the sum of statistical power estimates for each component study. It was also assumed that the power estimate of each component study depended on the effect size of the largest study (i.e., the smallest standard error) for each association [31,34]. When O > E and the *p*-value for the excess significance test < 0.10, excess significance was considered positive. The STATA version 16.0 (Stata Corporation, College Station, TX, USA) was used for all analyses.

### 2.5. Assessment of Evidence Credibility

Evidence credibility was assessed using the established criteria from previous umbrella reviews [35,36,37,38,39]. Associations with significant summary effect sizes (*p* < 0.05) were rated as four classifications: class I: convincing; class II: highly suggestive; class III: suggestive; class IV: weak.

For class I: *p* < 10^−6^, number of cases > 1000, *p* < 0.05 of the largest study, 95% PI excluding the null value, I^2^ < 50%, *p*-value for Egger’s test > 0.10, *p*-value for excess significance test > 0.10.

For class II: *p* < 10^−6^, number of cases > 1000, *p* < 0.05 of the largest study.

For class III: *p* < 10^−3^ and number of cases > 1000.

For class IV: the summary results of *p* < 0.05.

The summary results of *p* > 0.05 were ranked as class V with no significance.

## 3. Results

### 3.1. Literature Searches

**Figure 1:**
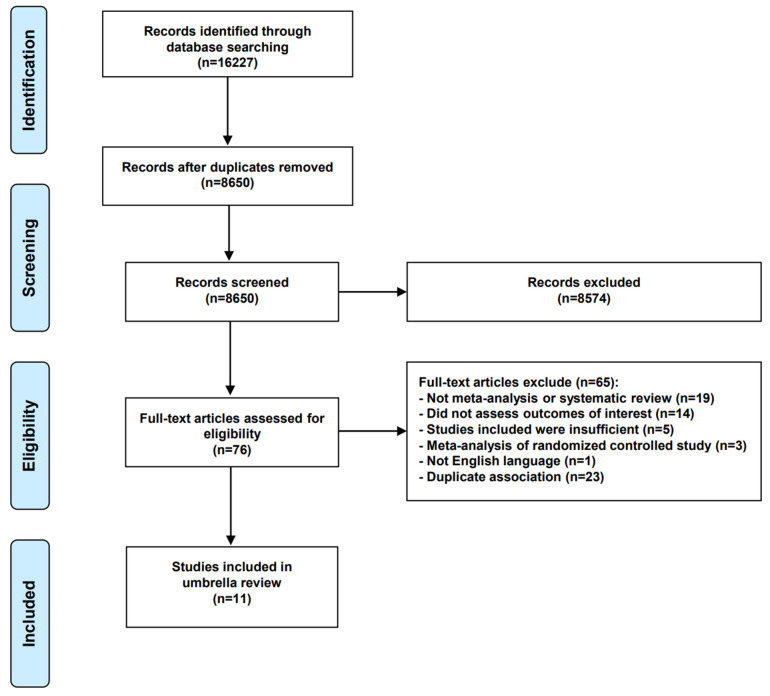
Literature search flow diagram.

A total of 16,227 studies were screened, and 76 full-text articles were evaluated for eligibility after removing duplicates and screening titles and abstracts. Ultimately, 11 meta-analyses were included in this umbrella review [40,41,42,43,44,45,46,47,48,49,50]. The study selection flow diagram is shown (Figure 1) and the reasons for excluding 65 articles (85.5%) are provided (Table S2).

### 3.2. Characteristics of Included Articles

**Figure 2:**
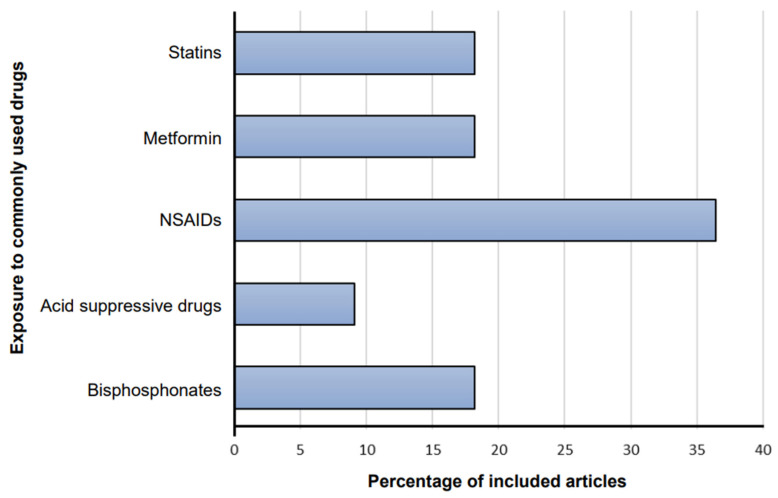
Distribution of included articles related to drug type. Abbreviations: NSAIDs, non-steroidal anti-inflammatory drugs.

19 associations were described in 11 eligible meta-analyses, including 147 individual study estimates of GC risk related to exposure to commonly used drugs. The characteristics of these 11 studies and their distributions related to drug type are shown (Table 1 and Figure 2). The included meta-analyses that were focused on associations of GC risk and its subtypes with any statins, atorvastatin, simvastatin, pravastatin, metformin, aspirin, NSAIDs, non-aspirin NSAIDs, acid suppressing drugs, histamine 2-receptor antagonists (H2RAs), PPIs, as well as any bisphosphonates and alendronates. In addition, three meta-analyses [40,44,46] included six associations between aspirin, NSAIDs, and non-aspirin NSAIDs with the risk of GC subtypes. All articles were published between 2009 and 2021, of which seven (63.6%) were published in the last five years. There were 3 to 32 study estimates combined with each meta-analysis, with a median of seven studies. The number of GC cases and total participants in meta-analyses was 67,866 and 19,505,291, respectively. The minimum number of cases in the meta-analysis was 387, but all except two (pravastatin and alendronate) had more than 1000 cases.

### 3.3. Methodological Quality Assessment

**Figure 3:**
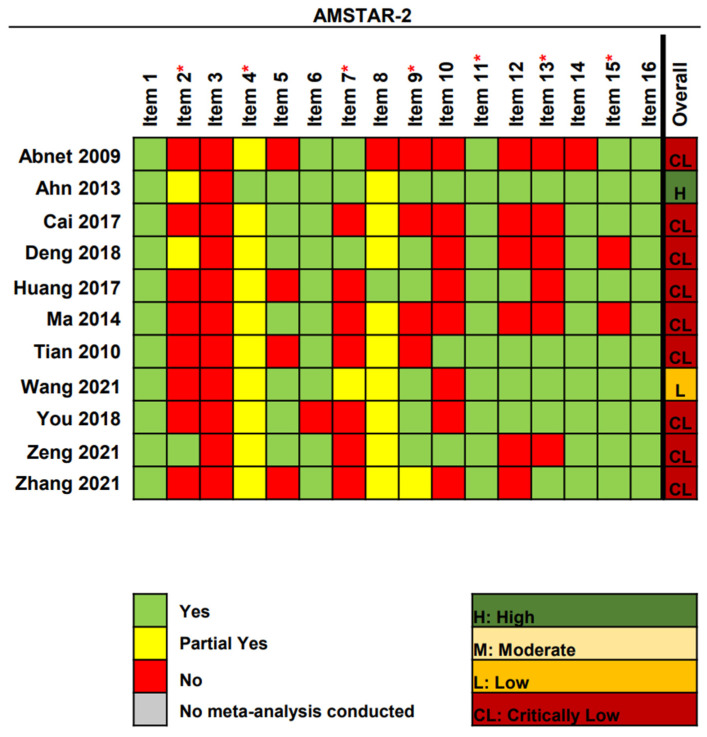
Methodological quality of the included systematic reviews [40,41,42,43,44,45,46,47,48,49,50]. Abbreviations: AMSTAR-2, A Measurement Tool to Assess Systematic Reviews-2; * Critical domain.

Using AMSTAR-2, only one (9.1%) meta-analysis was graded as high quality, one (9.1%) was low, and nine (81.8%) were critically low (Figure 3). All studies had more than one critical defect (typically in items 2 (72.7%), 7 (63.6%), and 13 (54.5%)) and several non-critical defects (typically in items 3 (100%), 10 (72.7%), and 12 (54.5%)). The rating criteria for methodological quality assessment are detailed (Table S3).

### 3.4. Summary Effect Size

The random-effect model was used to re-perform meta-analyses of 19 associations (Table 2). Twelve associations (63.2%) were statistically significant at *p* ≤ 0.05, and only one association (5.3%) between NSAIDs and GC risk reached *p* < 10^−6^. Moreover, four associations (21.1%) showed moderate statistical significance (*p* < 10^−3^), including non-aspirin NSAIDs and GC risk, NSAIDs and non-cardia GC risk, aspirin and non-cardia GC risk, as well as acid-suppressing drugs and GC risk. The majority of associations that reached statistical significance suggested potential preventative effects on GC risk, including statins, metformin, NSAIDs, aspirin, and non-aspirin NSAIDs. However, acid-suppressing drugs, including H2RAs and PPIs, increased the risk of GC. The effect of the largest study per association indicated that 7 of 19 were significant at *p* < 0.05.

### 3.5. Study Heterogeneity

Out of 19 associations, 15 (78.9%) showed significant heterogeneity. Besides, high heterogeneity (I^2^ > 75%) was observed in five associations (26.3%) with GC risk: atorvastatin, simvastatin, pravastatin, aspirin, and PPIs. The inter-study heterogeneity was analyzed by calculating the 95% PI value, and only one association (5.3%) between non-aspirin NSAIDs and GC risk excluded the null value (Table 2).

### 3.6. Small-Study Effects

The small-study effect was found in five associations (26.3%): any statins and GC risk, simvastatin and GC risk, NSAIDs and GC risk, NSAIDs and non-cardia GC risk, as well as non-aspirin NSAIDs and non-cardia GC risk (Table 2). However, only three associations (15.8%) included 10 or more studies, providing sufficient statistical power for Egger’s test to fully determine the small-study effect of NSAIDs, aspirin, and acid-suppressing drugs associated with GC risk.

### 3.7. Excess Significance

No evidence of excess significance bias was observed when the plausible effect size was supposed to be equal to the largest study estimate (Table 2).

### 3.8. Evidence Grading

**Figure 4:**
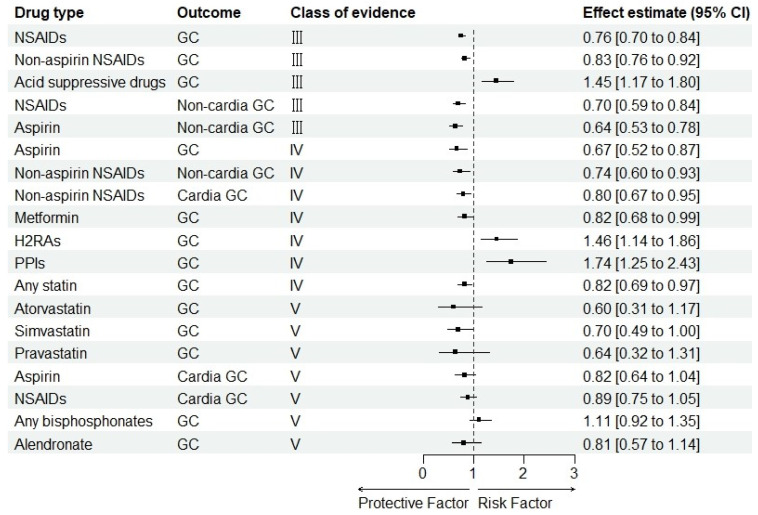
Summary estimates of commonly used drugs and GC risk by class of evidence. Abbreviations: GC, gastric cancer; NSAIDs, non-steroidal anti-inflammatory drugs; H2RAs, Histamine 2-receptor antagonist; PPIs, proton pump inhibitors; CI, confidence interval.

Of 19 associations between commonly used drugs and GC risk and its subtypes, no association was supported by convincing or highly suggestive evidence. Five associations (26.3%) were supported by suggestive evidence: the association between NSAIDs use, non-aspirin NSAIDs use and decreased GC risk, the association between NSAIDs use, aspirin use and decreased non-cardia GC risk, and the association between acid-suppressing drug use and increased GC risk (Table 2 and Figure 4). Notably, the meta-analysis indicated an association between acid-suppressing drug use and GC risk, attaining a high-quality level by AMSTAR-2 [41]. For the remaining associations, either weak evidence (36.8%) or non-significant evidence (36.8%) was found.

## 4. Discussion

### 4.1. Main Findings

Commonly used drugs may have potential links with various cancer types during the treatment of chronic diseases. To date, several systematic reviews or meta-analyses have explored associations between commonly used drugs and GC risk [51,52,53,54,55]. However, conclusions on relationships and methodological quality were inconsistent. Hence, the next step is to review and grade evidence from the existing systematic reviews and meta-analyses, thus providing an overview of the findings of a specific association. This umbrella review was therefore conducted.

In this study, two pieces of suggestive evidence showed that the intake of NSAIDs and non-aspirin NSAIDs could reduce the incidence of GC, while another one showed that the intake of acid-suppressing drugs was positively related to a higher risk of GC. For subtypes of GC, two pieces of suggestive evidence suggested that the increased intake of NSAIDs and aspirin was related to a lower risk of non-cardia GC. Five pieces of weak evidence suggested that the increased intake of aspirin, metformin and any statins was negatively associated with the incidence of GC, while the intake of H2RAs and PPIs was positively associated with a higher risk of GC. Two pieces of weak evidence showed that the increased intake of non-aspirin NSAIDs intake could have a negative correlation with the incidence of both cardia GC and non-cardia GC. The remaining drugs had no association with GC risk. All of these were previously summarized in Figure 4.

### 4.2. Comparison with Other Studies

NSAIDs, including aspirin and non-aspirin NASIDs, are commonly used to treat fever, pain, and inflammation. Recently, the anti-cancer effects of NSAIDs have also been reported in several cancer prevention studies [56,57,58]. However, a number of studies evaluating associations between NSAIDs and GC risk have been controversial [59,60]. This study found that the use of NSAIDs was related to a reduced risk of GC and non-cardia GC, which is consistent with a previous meta-analysis [44]. Moreover, aspirin is one of the world’s most prescribed NSAIDs. A cohort study in Korea showed that the long-term use of aspirin was negatively related to GC risk [59]. A large population-based case–control study concluded that the use of aspirin was inversely associated with the risk of GC and non-cardia GC [61]. Similarly, in this study, aspirin use lowered the risk of GC and non-cardia GC. In addition, it was found that the use of non-aspirin NSAIDs was negatively related to GC risk, which was also found in both cardia GC and non-cardia GC. Interestingly, one meta-analysis, including five clinical studies, explored the risk of non-aspirin NSAIDs and cardia GC [40]. All but one study reported no evidence that non-aspirin NSAIDs reduced the risk of cardia GC [62]. As for the risk of cardia GC associated with NSAIDs and aspirin, previous studies showed that aspirin or NSAIDs was not related to the risk of cardia GC [63,64,65], which is consistent with our findings. The anti-tumor mechanisms underpinning NSAIDs have not yet been fully elucidated. As inflammation is a critical component of tumor progression, a plausible explanation could be the anti-inflammatory effects of NASIDs [66]. Furthermore, the cyclooxygenase-2 (COX-2)/prostaglandin E2 (PGE2) pathway is associated with GC development, and PGE2 also plays a pro-inflammatory mediator role in GC development [67]. Thus, NSAIDs could reduce GC risk by inhibiting COX-2 expression [68].

Acid-suppressing drugs, including H2RAs and PPIs, are primary treatments for peptic ulcer disease, gastroesophageal reflux disease, and other digestive diseases [69,70]. This study observed that acid-suppressing drugs, either H2RAs or PPIs, could increase the risk of GC. One possible mechanism could be that acid-suppressing drugs increase gastrin secretion to reduce gastric acidity, thus leading to hypergastrinemia [71]. As a result, elevated serum gastrin may facilitate GC [72]. Recent studies also showed that these drugs were positively related to the risk of GC [73,74], whereas other studies provided evidence to the contrary. Lee et al. found that PPIs use was not related to an increased risk of GC [75]. Additionally, the results of a large prospective cohort suggested no association between H2RAs use and GC risk [15]. The inclusion of people from different regions may contribute to the inconsistency, and differences in the length of follow-up may be one of the reasons for the inconsistency.

In addition to PPIs, the role of statins and metformin in GC development has also been widely studied [76,77,78]. Statins reduce cholesterol and the incidence of cardiovascular disease [79]. Metformin is a first-line oral antidiabetic agent for the treatment of Type 2 diabetes [80]. Previous studies reported that statins and metformin exerted protective effects toward multiple cancers, including GC [81,82,83]. In this study, the increased intake of statins and metformin was found to be negatively correlated with GC risk. However, there was no negative relationship between any of three statin types (atorvastatin, simvastatin, and pravastatin) and GC risk, which is consistent with the findings of Vinogradova et al. [84]. Currently, there is also limited mechanistic evidence on how statins reduce the incidence of GC. One possible mechanism could be that statins evoke apoptosis and anti-angiogenesis [85]. The underlying anti-tumor mechanism of metformin is to activate the 5′ adenosine monophosphate-activated protein kinase pathway [86].

### 4.3. Limitations

This is the first umbrella review to systematically explore evaluate associations between commonly used drugs and the risk of GC and its subtypes through published systematic reviews and meta-analyses of observational studies. However, there are several limitations to this study. First, the evidence from meta-analyses of observational studies was graded only. Observational studies often have more potential for bias and confounding issues when compared with randomized controlled studies. Thus, the associations identified in observational studies did not necessarily imply causality. Second, the credibility of the umbrella review depended directly on meta-analyses and indirectly on individual studies; therefore, some biases were inevitable. Third, the methodological quality of most systematic reviews and meta-analyses was critically low from the perspective of AMSTAR-2 analyses. Fourth, drug use and the risk of GC subtypes was not comprehensively reviewed due to a lack of data. For example, associations between acid-suppressing drugs and the risk of GC subtypes were not reviewed because the number of GC subtypes from an individual study was not reported [87]. Furthermore, drug dose, frequency, and duration of drug use and GC risk were not evaluated due to data limitations.

## 5. Conclusions

To conclude, three associations between GC risk and commonly used drugs (NSAIDs, non-aspirin NSAIDs, and acid-suppressing drugs) were supported by suggestive evidence. It was found that the intake of NSAIDs or non-aspirin NSAIDs could reduce GC risk, whereas the intake of acid-suppressing drugs could increase GC risk. Moreover, the decreased risk of non-cardia GC associated with NSAIDs or aspirin was also supported by suggestive evidence. However, caution should be exercised when interpreting these relationships, and high-quality studies are required in the future to determine whether these associations have absolute causality.

## Figures and Tables

**Table 1:** Characteristics of the associations in the included systematic reviews and meta-analyses.

First Author [Ref], Year	Country	Drug Type	Outcome	Comparison	No. of Included Studies	No. of Cases/Population
You [48], 2018	China	Any statins	GC	Any users vs. never users	8	3365/7,394,525
Ma [45], 2014	China	Atorvastatin	GC	Any users vs. never users	3	1580/22,476
	China	Simvastatin	GC	Any users vs. never users	3	1757/22,476
	China	Pravastatin	GC	Any users vs. never users	3	463/22,476
Wang [47], 2021	China	Aspirin	GC	Regular users vs. never users	10	14,933/2,378,794
Huang [44], 2017	China	NSAIDs	GC	Any users vs. never users	32	9568/2,633,756
	China	NSAIDs	Non-cardia GC	Any users vs. never users	8	2772/488,590
	China	Aspirin	Non-cardia GC	Any users vs. never users	7	2332/487,375
	China	Non-aspirin NSAIDs	Non-cardia GC	Any users vs. never users	5	1507/484,760
Abnet [40], 2009	United States	Aspirin	Cardia GC	Any users vs. never users	7	1467/318,431
	United States	Non-aspirin NSAIDs	Cardia GC	Any users vs. never users	5	1303/316,734
Tian [46], 2010	China	Non-aspirin NSAIDs	GC	Ever users vs. never users	5	3145/328,258
	China	NSAIDs	Cardia GC	Ever users vs. never users	5	1036/315,596
Zhang [50], 2021	China	Metformin	GC	Ever users vs. never users	7	2253/1,136,484
Ahn [41], 2013	South Korea	Acid suppressive drugs	GC	Any users vs. never users	10	4628/49,363
	South Korea	H2RAs	GC	Any users vs. never users	9	3409/41,432
Zeng [49], 2021	China	PPIs	GC	Any users vs. never users	9	7071/2,344,365
Cai [42], 2017	China	Any bisphosphonates	GC	Any users vs. never users	8	4890/516,849
Deng [43], 2018	China	Alendronate	GC	Any users vs. never users	3	387/202,551

Abbreviations: GC, gastric cancer; NSAIDs, non-steroidal anti-inflammatory drugs; H2RAs, Histamine 2-receptor antagonist; PPIs, proton pump inhibitors.

**Table 2:** Evidence-rating results on associations between commonly used drugs and the risk of gastric cancer.

First Author [Ref], Year	Drug Type	Outcome	Random-Effects Summary Effect Size (95% CI)	Random *p* Value	I^2^	95% PI	Egger *p* Value	LS	*p* Value *	Influential Factors	CE
You [48], 2018	Any statins	GC	0.82 (0.69–0.97)	0.019	69.5%	(0.52–1.29)	0.041	No	0.476	Protective factor	IV
Ma [45], 2014	Atorvastatin	GC	0.60 (0.31–1.17)	0.136	96.2%	(0.00–3080.40)	0.198	No	NP	NA	V
	Simvastatin	GC	0.70 (0.49–1.00)	0.052	85.6%	(0.01–49.50)	0.077	No	1.000	NA	V
	Pravastatin	GC	0.64 (0.32–1.31)	0.221	88.0%	(0.00–3939.07)	0.180	No	NP	NA	V
Wang [47], 2021	Aspirin	GC	0.67 (0.52–0.87)	0.003	96.2%	(0.26–1.76)	0.531	No	0.764	Protective factor	IV
Huang [44], 2017	NSAIDs	GC	0.76 (0.70–0.84)	5.72 × 10^−9^	64.8%	(0.51–1.14)	0.034	No	0.143	Protective factor	III
	NSAIDs	Non-cardia GC	0.70 (0.59–0.84)	1.33 × 10^−4^	69.4%	(0.40–1.24)	0.054	Yes	0.139	Protective factor	III
	Aspirin	Non-cardia GC	0.64 (0.53–0.78)	9.60 × 10^−6^	69.2%	(0.35–1.18)	0.105	Yes	0.111	Protective factor	III
	Non-aspirin NSAIDs	Non-cardia GC	0.74 (0.60–0.93)	8.58 × 10^−3^	58.8%	(0.37–1.50)	0.023	No	0.687	Protective factor	IV
Abnet [40], 2009	Aspirin	Cardia GC	0.82 (0.64–1.04)	0.099	68.1%	(0.39–1.70)	0.653	No	NP	NA	V
	Non-aspirin NSAIDs	Cardia GC	0.80 (0.67–0.95)	0.013	35.6%	(0.49–1.29)	0.195	Yes	NP	Protective factor	IV
Tian [46], 2010	Non-aspirin NSAIDs	GC	0.83 (0.76–0.92)	1.53 × 10^−4^	0.0%	(0.72–0.97)	0.118	No	0.181	Protective factor	III
	NSAIDs	Cardia GC	0.89 (0.75–1.05)	0.167	28.3%	(0.58–1.36)	0.313	No	1.000	NA	V
Zhang [50], 2021	Metformin	GC	0.82 (0.68–0.99)	0.037	73.2%	(0.48–1.41)	0.914	Yes	NP	Protective factor	IV
Ahn [41], 2013	Acid suppressive drugs	GC	1.45 (1.17–1.80)	7.07 × 10^−4^	53.8%	(0.82–2.56)	0.819	Yes	0.289	Risk factor	III
	H2RAs	GC	1.46 (1.14–1.86)	2.51 × 10^−3^	60.3%	(0.74–2.85)	0.699	Yes	0.289	Risk factor	IV
Zeng [49], 2021	PPIs	GC	1.74 (1.25–2.43)	1.14 × 10^−3^	94.0%	(0.53–5.71)	0.186	Yes	NP	Risk factor	IV
Cai [42], 2017	Any bisphosphonates	GC	1.11 (0.92–1.35)	0.266	59.1%	(0.64–1.93)	0.407	No	0.489	NA	V
Deng [43], 2018	Alendronate	GC	0.81 (0.57–1.14)	0.229	39.0%	(0.03–22.61)	0.631	No	1.000	NA	V

Abbreviations: GC, gastric cancer; NSAIDs, non-steroidal anti-inflammatory drugs; H2RAs, Histamine 2-receptor antagonist; PPIs, proton pump inhibitors; CI, confidence interval; PI, prediction interval; LS, largest study with significant effect; CE, class of evidence; NP, not pertinent because of fewer-than-expected number of observed studies; NA, not applicable. * *p* value of excess significance test. All statistical tests two sided.

## Supplementary Materials

The following supporting information can be downloaded at: https://www.mdpi.com/article/10.3390/cancers15020372/s1, Table S1: Search strategy; Table S2: The list of excluded studies; Table S3: Rating criteria for methodological quality assessment. References [40,41,42,43,44,45,46,47,48,49,50,88,89,90,91,92,93,94,95,96,97,98,99,100,101,102,103,104,105,106,107,108,109,110,111,112,113,114,115,116,117,118,119,120,121,122,123,124,125,126,127,128,129,130,131,132,133,134,135,136,137,138,139,140,141,142,143,144,145,146] are cited in the Supplementary Materials.
