# Supplementary material for: Exposure to Commonly Used Drugs and the Risk of Gastric Cancer: An Umbrella Review of Meta-Analyses
Source: Cancers (Basel). 2023 Jan 6;15(2):372. doi: 10.3390/cancers15020372 (PMC9856677; doi:10.3390/cancers15020372)
Supplement: Supplementary file 1 [file cancers-15-00372-s001.zip › cancers-2038198-supplementary.pdf]

cancers

MDPI

Supplementary Materials: Exposure to Commonly Used Drugs and the Risk of Gastric Cancer: An Umbrella Review of Meta-Analyses

Xiao Bai [1], Si-Qi Ding [1], Xue-Ping Zhang [1], Ming-Hao Han [1] and Dong-Qiu Dai [1,2,*]

Table S1. Search strategy

PubMed: 4532<br><br>(((((((((((("gastric cancer") OR ("gastric carcinoma")) OR ("gastric neoplasm")) OR ("gastric tumor")) OR ("gastric neoplasia")) OR ("gastric malignancy")) OR ("GC")) OR ("stomach neoplasm")) OR ("stomach cancer")) OR ("stomach carcinoma")) OR ("stomach tumor")) OR ("stomach neoplasia")) OR ("stomach malignancy")) AND ((meta) OR (meta-analysis))
Web of Science: 5625<br><br>1: TS= ("gastric cancer" OR "gastric carcinoma" OR "gastric neoplasm" OR "gastric tumor" OR "gastric neoplasia" OR "gastric malignancy" OR "stomach neoplasm" OR "stomach cancer" OR "stomach carcinoma" OR "stomach tumor" OR "stomach neoplasia" OR "stomach malignancy" OR "GC")<br>2: TS= (meta OR meta-analysis)<br>3: 1 AND 2
EMBASE: 6070<br><br>1: 'gastric cancer' OR 'gastric carcinoma' OR 'gastric neoplasm' OR 'gastric tumor' OR 'gastric neoplasia' OR 'gastric malignancy' OR 'stomach neoplasm' OR 'stomach cancer' OR 'stomach carcinoma' OR 'stomach tumor' OR 'stomach neoplasia' OR 'stomach malignancy' OR 'GC'<br>2: meta OR 'meta analysis'<br>3: 1 and 2

Table S2. The list of excluded studies

Study	Reasons for exclusion
Wang et al. 2021[1]	no incidence of gastric cancer
Sun et al. 2021 [2]	not meta-analysis or systematic review
Sun et al. 2021 [3]	no incidence of gastric cancer
Song et al. 2021 [4]	no incidence of gastric cancer
Seo et al. 2021[5]	duplicate association
Segna et al. 2021[6]	duplicate association
Goyal et al. 2021 [7]	no incidence of gastric cancer
Zhou et al. 2020 [8]	no incidence of gastric cancer
Zheng et al. 2020 [9]	no incidence of gastric cancer
Win et al. 2020 [10]	duplicate association
Thomas et al. 2020 [11]	meta-analysis of randomized controlled study
Song et al, 2020 [12]	duplicate association
Shuai et al. 2020 [13]	duplicate association
Segna et al. 2020 [14]	not meta-analysis or systematic review
Niikura et al. 2020 [15]	duplicate association
Lin et al. 2020 [16]	no incidence of gastric cancer
Indini et al. 2020 [17]	no incidence of gastric cancer
Bosetti et al. 2020 [18]	duplicate association
Bindu et al. 2020 [19]	not meta-analysis or systematic review
Abdel-Rahman et al. 2020 [20]	not meta-analysis or systematic review
Wan et al. 2019 [21]	not meta-analysis or systematic review
Jiang et al. 2019 [22]	duplicate association
Huang et al. 2019 [23]	not meta-analysis or systematic review
Cheung et al.2019 [24]	not meta-analysis or systematic review

Bao et al. 2019 [25]	duplicate association
Qiao et al. 2018 [26]	duplicate association
Jang et al. 2018 [27]	meta-analysis of randomized controlled study
Farooqi et al. 2018 [28]	meta-analysis of randomized controlled study
Zhou et al. 2017 [29]	duplicate association
Zhang et al. 2017 [30]	included less than 3 studies
Tran-Duy et al. 2017 [31]	not meta-analysis or systematic review
Palmer et al. 2017 [32]	not meta-analysis or systematic review
Mei et al. 2017 [33]	no incidence of gastric cancer
Long et al. 2017 [34]	not English language
Kim et al 2017 [35]	included less than 3 studies
Kamal et al. 2017 [36]	not meta-analysis or systematic review
Joo et al. 2017 [37]	not meta-analysis or systematic review
Wang et al. 2016 [38]	not meta-analysis or systematic review
Tran-Duy et al. 2016 [39]	duplicate association
Kong et al. 2016 [40]	duplicate association
Wright et al. 2015 [41]	duplicate association
Stryjkowska-Gora et al. 2015 [42]	not meta-analysis or systematic review
Stegeman et al. 2015 [43]	not meta-analysis or systematic review
Huang et al. 2015 [44]	not meta-analysis or systematic review
Vallianou et al. 2014 [45]	not meta-analysis or systematic review
Song et al. 2014 [46]	no incidence of gastric cancer
Ye et al. 2013 [47]	duplicate association
Wu et al. 2013 [48]	duplicate association
Singh et al. 2013 [49]	duplicate association
Malek et al. 2013 [50]	not meta-analysis or systematic review

Franciosi et al. 2013 [51]	included less than 3 studies
Eslami et al. 2013 [52]	no incidence of gastric cancer
Oh et al. 2012 [53]	duplicate association
Colmers et al. 2012 [54]	no incidence of gastric cancer
Bosetti et al. 2012 [55]	duplicate association
Algra et al. 2012 [56]	duplicate association
Rothwell et al. 2011 [57]	no incidence of gastric cancer
Yang et al. 2010 [58]	duplicate association
Matsushita et al. 2010 [59]	no incidence of gastric cancer
Kuoppala et al.2008 [60]	included less than 3 studies
Browning et al. 2007 [61]	included less than 3 studies
Wang et al. 2003 [62]	duplicate association
Gonzalez-Perez et al. 2003 [63]	duplicate association
Jolly et al. 2002 [64]	not meta-analysis or systematic review
Bosetti et al. 2002 [65]	not meta-analysis or systematic review

Table S3. Rating criteria for methodological quality assessment

References	1	2*	3	4*	5	6	7*	8	9*	10	11*	12	13*	14	15*	16	Rating
Abnet et al. 2009 [66]	Yes	No	No	PY	No	Yes	Yes	No	No	No	Yes	No	No	No	Yes	Yes	CL
Ahn et al. 2013 [67]	Yes	PY	No	Yes	Yes	Yes	Yes	PY	Yes	Yes	Yes	Yes	Yes	Yes	Yes	Yes	H
Cai et al. 2017 [68]	Yes	No	No	PY	Yes	Yes	No	PY	No	No	Yes	No	No	Yes	Yes	Yes	CL
Deng et al. 2018 [69]	Yes	PY	No	PY	Yes	Yes	Yes	PY	Yes	No	Yes	No	No	Yes	No	Yes	CL
Huang et al. 2017 [70]	Yes	No	No	PY	No	Yes	No	Yes	Yes	No	Yes	Yes	No	Yes	Yes	Yes	CL
Ma et al. 2014 [71]	Yes	No	No	PY	Yes	Yes	No	PY	No	No	Yes	No	No	Yes	No	Yes	CL
Tian et al. 2010 [72]	Yes	No	No	PY	No	Yes	No	PY	No	Yes	Yes	Yes	Yes	Yes	Yes	Yes	CL
Wang et al. 2021 [73]	Yes	No	No	PY	Yes	Yes	PY	PY	Yes	No	Yes	Yes	Yes	Yes	Yes	Yes	L
You et al. 2018 [74]	Yes	No	No	PY	Yes	No	No	PY	Yes	No	Yes	Yes	Yes	Yes	Yes	Yes	CL
Zeng et al. 2021 [75]	Yes	Yes	No	PY	Yes	Yes	No	PY	Yes	Yes	Yes	No	No	Yes	Yes	Yes	CL
Zhang et al. 2021 [76]	Yes	No	No	PY	No	Yes	No	PY	PY	No	Yes	No	Yes	Yes	Yes	Yes	CL

Abbreviations: PY: partial yes; H: high; L: low; CL: critically low; * critical domains.

AMSTAR Checklist 2: (*critical domains)

1. Did the research questions and inclusion criteria for the review include the components of PICO?

The research questions and inclusion criteria for the review should include Population, Intervention, Comparator group, and Outcome.

Note: Timeframe for follow-up is optional (recommended) to get a yes.

2. Did the report of the review contain an explicit statement that the review methods were established prior to the conduct of the review and did the report justify any significant deviations from the protocol? *

For Partial Yes:

The authors state that they had a written protocol or guide that included ALL the following: review question(s), a search strategy, inclusion/exclusion criteria, a risk

of bias assessment.

For Yes:

As for partial yes, plus the protocol should be registered and should also have specified: a meta-analysis/synthesis plan, if appropriate, and a plan for investigating causes of heterogeneity, justification for any deviations from the protocol.

3. Did the review authors explain their selection of the study designs for inclusion in the review?

For Yes, the review should satisfy ONE of the following: explanation for including only RCTs, OR explanation for including only NRSI, OR explanation for including both RCTs and NRSI.

4. Did the review authors use a comprehensive literature search strategy? *

For Partial Yes (ALL the following): searched at least 2 databases (relevant to research question), provided key word and/or search strategy, justified publication restrictions (eg, language). For Yes, should also have (all the following): searched the reference lists/bibliographies of included studies, searched trial/study registries, included/consulted content experts in the field, where relevant, searched for grey literature, conducted search within 24 months of completion of the review.

5. Did the review authors perform study selection in duplicate?

For Yes, either ONE of the following: at least two reviewers independently agreed on selection of eligible studies and achieved consensus on which studies to include, OR two reviewers selected a sample of eligible studies and achieved good agreement (at least 80 per cent), with the remainder selected by one reviewer.

6. Did the review authors perform data extraction in duplicate?

For Yes, either ONE of the following: at least two reviewers achieved consensus on which data to extract from included studies, OR two reviewers extracted data from a sample of eligible studies and achieved good agreement (at least 80 per cent), with the remainder extracted by one reviewer.

7. Did the review authors provide a list of excluded studies and justify the exclusions? *

For Partial Yes: provided a list of all potentially relevant studies that were read in full text form but excluded from the review

For Yes, must also have: justified the exclusion from the review of each potentially relevant study.

8. Did the review authors describe the included studies in adequate detail?

For Partial Yes (ALL the following): described populations, described interventions, described comparators, described outcomes, described research designs.

For Yes, should also have ALL the following: described population in detail, described intervention and comparator in detail (including doses where relevant),

described study's setting, timeframe for follow-up.

9. Did the review authors use a satisfactory technique for assessing the risk of bias (RoB) in individual studies that were included in the review? *

RCTs

For Partial Yes, must have assessed RoB from unconcealed allocation, and lack of blinding of patients and assessors when assessing outcomes (unnecessary for objective outcomes such as all-cause mortality).

For Yes, must also have assessed RoB from: allocation sequence that was not truly random, and selection of the reported result from among multiple measurements or analyses of a specified outcome.

NRSI

For Partial Yes, must have assessed RoB: from confounding, and from selection bias.

For Yes, must also have assessed RoB: methods used to ascertain exposures and outcomes, and selection of the reported result from among multiple measurements or analyses of a specified outcome.

10. Did the review authors report on the sources of funding for the studies included in the review?

For Yes, must have reported on the sources of funding for individual studies included in the review. Note: Reporting that the reviewers looked for this information but it was not reported by study authors also qualifies.

11. If meta-analysis was performed did the review authors use appropriate methods for statistical combination of results? *

RCTs

For Yes: the authors justified combining the data in a meta-analysis, AND they used an appropriate weighted technique to combine study results and adjusted for heterogeneity if present, AND investigated the causes of any heterogeneity.

NRSI

For Yes: the authors justified combining the data in a meta-analysis, AND they used an appropriate weighted technique to combine study results, adjusting for heterogeneity if present, AND they statistically combined effect estimates from NRSI that were adjusted for confounding, rather than combining raw data, or justified combining raw data when adjusted effect estimates were not available, AND they reported separate summary estimates for RCTs and NRSI separately when both were included in the review.

12. If meta-analysis was performed, did the review authors assess the potential impact of RoB in individual studies on the results of the meta-analysis or other evidence synthesis?

For Yes: included only low risk of bias RCTs, OR, if the pooled estimate was based on RCTs and/or NRSI at variable RoB, the authors performed analyses to investigate possible impact of RoB on summary estimates of effect.

13. Did the review authors account for RoB in individual studies when interpreting/discussing the results of the review? *

For Yes: included only low risk of bias RCTs, OR, if RCTs with moderate or high RoB, or NRSI were included the review provided a discussion of the likely impact of RoB on the results.

14. Did the review authors provide a satisfactory explanation for, and discussion of, any heterogeneity observed in the results of the review?

For Yes: there was no significant heterogeneity in the results, OR if heterogeneity was present the authors performed an investigation of sources of any heterogeneity in the results and discussed the impact of this on the results of the review.

15. If they performed quantitative synthesis did the review authors carry out an adequate investigation of publication bias (small study bias) and discuss its likely impact on the results of the review? *

For Yes: performed graphical or statistical tests for publication bias and discussed the likelihood and magnitude of impact of publication bias.

16. Did the review authors report any potential sources of conflict of interest, including any funding they received for conducting the review?

For Yes: the authors reported no competing interests, OR the authors described their funding sources and how they managed potential conflicts of interest.

Rating overall confidence in the results of the review

High: No or one non-critical weakness: the systematic review provides an accurate and comprehensive summary of the results of the available studies that address the question of interest.

Moderate: More than one non-critical weakness*: the systematic review has more than one weakness but no critical flaws. It may provide an accurate summary of the results of the available studies that were included in the review.

Low: One critical flaw with or without non-critical weaknesses: the review has a critical flaw and may not provide an accurate and comprehensive summary of the available studies that address the question of interest.

Critically low: More than one critical flaw with or without non-critical weaknesses: the review has more than one critical flaw and should not be relied on to provide an accurate and comprehensive summary of the available studies.

References

1. Wang, X.; Luo, Y.; Chen, T.; Zhang, K. Low-dose aspirin use and cancer-specific mortality: a meta-analysis of cohort studies. Journal of public health (Oxford, England) 2021, 43, 308-315, doi:10.1093/pubmed/fdz114.
2. Sun, C.; Chen, Y.; Ismail, M.R.; Tuason, J.P.W.; Cheng, X.; Hu, L.; Bhan, C.; Kim, N.H.; Prasad, A.; Manem, N.; et al. Is Acid Suppression Therapy Associated With Increased Risk of Cardia Gastric Cancer? A Meta-Analysis. American Journal of Gastroenterology 2021, 116, S655-S655.
3. Sun, C.; Tuason, J.P.W.; Kim, K.Y.; Cheng, C.; Bhan, C.; Manem, R.; Sundararajan, N.; Gerais, Y.A.; Gandam, M.R.; Lising, J.F.; et al. Is Metformin Associated With Decreased Mortality of Gastric Cancer? A Meta-Analysis. American Journal of Gastroenterology 2021, 116, S637-S638.
4. Song, H.J.; Rhew, K.; Lee, Y.J.; Ha, I.-H. Acid-suppressive agents and survival outcomes in patients with cancer: a systematic review and meta-analysis. International Journal of Clinical Oncology 2021, 26, 34-50, doi:10.1007/s10147-020-01795-7.
5. Seo, S.I.; Park, C.H.; Kim, T.J.; Bang, C.S.; Kim, J.Y.; Lee, K.J.; Kim, J.; Kim, H.H.; You, S.C.; Shin, W.G. Aspirin, metformin, and statin use on the risk of gastric cancer: A nationwide population-based cohort study in Korea with systematic review and meta-analysis. Cancer Medicine 2021, doi:10.1002/cam4.4514.
6. Segna, D.; Brusselaers, N.; Glaus, D.; Krupka, N.; Misselwitz, B. Association between proton-pump inhibitors and the risk of gastric cancer: a systematic review with meta-analysis. Therapeutic Advances in Gastroenterology 2021, 14, doi:10.1177/17562848211051463.
7. Goyal, H.; Sachdeva, S.; Perisetti, A.; Aloysius, M.M.; Chandan, S.; Tharian, B.; Thosani, N. Continued Aspirin Use and Bleeding Risk After Endoscopic Submucosal Dissection of Gastric Neoplasms: A Meta-Analysis. American Journal of Gastroenterology 2021, 116, S473-S474.
8. Zhou, Q.; Chen, D.-S.; Xin, L.; Zhou, L.-Q.; Zhang, H.-T.; Liu, L.; Yuan, Y.-W.; Li, S.-H. The renin-angiotensin system blockers and survival in digestive system malignancies: A systematic review and meta-analysis. Medicine 2020, 99, doi:10.1097/md.0000000000019075.
9. Zheng, J.; He, J.; Wang, W.; Zhou, H.; Cai, S.; Zhu, L.; Qian, X.; Wang, J.; Lu, Z.; Huang, C. The impact of pain and opioids use on survival in cancer patients: Results from a population-based cohort study and a meta-analysis. Medicine (United States) 2020, 99, doi:10.1097/MD.0000000000019306.
10. Win, T.T.; Aye, S.N.; Fern, J.L.C.; Fei, C.O. Aspirin and Reducing Risk of Gastric Cancer: Systematic Review and Meta-Analysis of the Observational Studies. Journal of Gastrointestinal and Liver Diseases 2020, 29, 191-198, doi:10.15403/jgld-818.
11. Thomas, J.P.; Loke, Y.K.; Alexandre, L. Efficacy and safety profile of statins in patients with cancer: a systematic review of randomised controlled trials. European Journal of Clinical Pharmacology 2020, 76, 1639-1651, doi:10.1007/s00228-020-02967-0.
12. Song, H.J.; Jeon, N.; Squires, P. The association between acid-suppressive agent use and the risk of cancer: a systematic review and meta-analysis. European Journal of Clinical Pharmacology 2020, 76, 1437-1456, doi:10.1007/s00228-020-02927-8.
13. Shuai, Y.; Li, C.; Zhou, X. The effect of metformin on gastric cancer in patients with type 2 diabetes: a systematic review and meta-analysis. Clinical & Translational Oncology 2020, 22, 1580-1590, doi:10.1007/s12094-020-02304-y.
14. Segna, D.; Brusselaers, N.; Glaus, D.; Krupka, N.; Misselwitz, B. Association between long-term use of proton pump inhibitors and the risk of gastric cancer: A systematic review and meta-analysis. United European Gastroenterology Journal 2020, 8, 227, doi:10.1177/2050640620927345.
15. Niikura, R.; Hirata, Y.; Hayakawa, Y.; Kawahara, T.; Yamada, A.; Koike, K. Effect of aspirin use on gastric cancer incidence and survival: A systematic review and meta-analysis. Jgh Open 2020, 4, 117-125, doi:10.1002/jgh3.12226.
16. Lin, J.-L.; Lin, J.-X.; Zheng, C.-H.; Li, P.; Xie, J.-W.; Wang, J.-b.; Lu, J.; Chen, Q.-Y.; Cao, L.-l.; Lin, M.; et al. Relationship between aspirin use of esophageal, gastric and colorectal cancer patient survival: a meta-analysis. Bmc Cancer 2020, 20, doi:10.1186/s12885-020-07117-4.

17. Indini, A.; Petrelli, F.; Tomasello, G.; Rijavec, E.; Facciorusso, A.; Grossi, F.; Ghidini, M. Impact of Use of Gastric-Acid Suppressants and Oral Anti-Cancer Agents on Survival Outcomes: A Systematic Review and Meta-Analysis. Cancers 2020, 12, doi:10.3390/cancers12040998.
18. Bosetti, C.; Santucci, C.; Gallus, S.; Martinetti, M.; La Vecchia, C. Aspirin and the risk of colorectal and other digestive tract cancers: an updated meta-analysis through 2019. Annals of Oncology 2020, 31, 558-568, doi:10.1016/j.annonc.2020.02.012.
19. Bindu, S.; Mazumder, S.; Bandyopadhyay, U. Non-steroidal anti-inflammatory drugs (NSAIDs) and organ damage: A current perspective. Biochemical Pharmacology 2020, 180, doi:10.1016/j.bcp.2020.114147.
20. Abdel-Rahman, O.; Karachiwala, H.; Easaw, J.C. Outcomes of advanced gastrointestinal (GI) cancer patients in relationship to opioid use: An individual patient data pooled analysis from eight clinical trials. Journal of Clinical Oncology 2020, 38, doi:10.1200/JCO.2020.38.4_suppl.687.
21. Wan, Q.-Y.; Wu, X.-T.; Li, N.; Du, L.; Zhou, Y. Long-term proton pump inhibitors use and risk of gastric cancer: a meta-analysis of 926 386 participants. Gut 2019, 68, doi:10.1136/gutjnl-2018-316416.
22. Jiang, K.; Jiang, X.; Wen, Y.; Liao, L.; Liu, F.B. Relationship between long-term use of proton pump inhibitors and risk of gastric cancer: A systematic analysis. Journal of Gastroenterology and Hepatology (Australia) 2019, 34, 1898-1905, doi:10.1111/jgh.14759.
23. Huang, C.; Lin, J.; Lin, J.; Zheng, C.; Li, P.; Xie, J.; Wang, J.; Lu, J.; Chen, Q.; Cao, L.; et al. Long-term use of proton pump inhibitors may increase the incidence of non-cardiac gastric cancer. Surgical Endoscopy 2019, 33, S754, doi:10.1007/s00464-019-07109-x.
24. Cheung, K.S.; Leung, W.K. Long-term use of proton-pump inhibitors and risk of gastric cancer: a review of the current evidence. Therapeutic Advances in Gastroenterology 2019, 12, doi:10.1177/1756284819834511.
25. Bao, C.; Wang, K.; Ding, Y.; Kong, J. Association Between Anti-bacterial Drug Use and Digestive System Neoplasms: A Systematic Review and Meta-analysis. Frontiers in Oncology 2019, 9, doi:10.3389/fonc.2019.01298.
26. Qiao, Y.; Yang, T.; Gan, Y.; Li, W.; Wang, C.; Gong, Y.; Lu, Z. Associations between aspirin use and the risk of cancers: a meta-analysis of observational studies. Bmc Cancer 2018, 18, doi:10.1186/s12885-018-4156-5.
27. Jang, H.J.; Kim, H.S.; Kim, J.H.; Lee, J. The effect of statin added to systemic anticancer therapy: A meta-analysis of randomized, controlled trials. Journal of Clinical Medicine 2018, 7, doi:10.3390/JCM7100325.
28. Farooqi, M.A.M.; Malhotra, N.; Mukherjee, S.D.; Sanger, S.; Dhesy-Thind, S.K.; Ellis, P.; Leong, D.P. Statin therapy in the treatment of active cancer: A systematic review and meta-analysis of randomized controlled trials. PLoS ONE 2018, 13, doi:10.1371/journal.pone.0209486.
29. Zhou, X.-L.; Xue, W.-H.; Ding, X.-F.; Li, L.-F.; Dou, M.-M.; Zhang, W.-J.; Lv, Z.; Fan, Z.-R.; Zhao, J.; Wang, L.-X. Association between metformin and the risk of gastric cancer in patients with type 2 diabetes mellitus: a meta-analysis of cohort studies. Oncotarget 2017, 8, 55622-55631, doi:10.18632/oncotarget.16973.
30. Zhang, T.; Yang, X.; Zhou, J.; Liu, P.; Wang, H.; Li, A.; Zhou, Y. Benzodiazepine drug use and cancer risk: a dose-response meta analysis of prospective cohort studies. Oncotarget 2017, 8, 102381-102391, doi:10.18632/oncotarget.22057.
31. Tran-Duy, A.; Spaetgens, B.; Hoes, A.W.; De Wit, N.J.; Stehouwer, C.D.A. Use of Proton Pump Inhibitors and Risks of Fundic Gland Polyps and Gastric Cancer: Systematic Review and Meta-analysis Reply. Clinical Gastroenterology and Hepatology 2017, 15, 790-790, doi:10.1016/j.cgh.2017.01.008.
32. Palmer, R.H. Use of Proton Pump Inhibitors and Risks of Fundic Gland Polyps and Gastric Cancer: Systematic Review and Meta-analysis. Clinical Gastroenterology and Hepatology 2017, 15, 790-790, doi:10.1016/j.cgh.2016.12.007.
33. Mei, Z.; Liang, M.; Li, L.; Zhang, Y.; Wang, Q.; Yang, W. Effects of statins on cancer mortality and progression: A systematic review and meta-analysis of 95 cohorts including 1,111,407 individuals. International Journal of Cancer 2017, 140, 1068-1081, doi:10.1002/ijc.30526.
34. Long, L.; Cao, G.; Li, Y.; Tang, S. Association of ACEIs/ARBs therapy with digestive system neoplasms: A meta-analysis. Chinese Journal of Evidence-Based Medicine 2017, 17, 1051-1059, doi:10.7507/1672-2531.201703036.

35. Kim, H.B.; Myung, S.K.; Park, Y.C.; Park, B. Use of benzodiazepine and risk of cancer: A meta-analysis of observational studies. International Journal of Cancer 2017, 140, 513-525, doi:10.1002/ijc.30443.
36. Kamal, F.; Khan, M.A.; Akbar, H.; Haq, K.F.; Cholankeril, G.; Hammad, T.A.; Ali, B.; Ismail, M.K.; Satapathy, S.K.; Howden, C.W. METFORMIN DOES NOT REDUCE THE RISK OF GASTRIC CANCER IN TYPE 2 DIABETICS: SYSTEMATIC REVIEW AND META-ANALYSIS. Gastroenterology 2017, 152, S336-S337, doi:10.1016/s0016-5085(17)31389-6.
37. Joo, M.K.; Park, J.-J.; Chun, H.J. Additional Benefits of Routine Drugs on Gastrointestinal Cancer: Statins, Metformin, and Proton Pump Inhibitors. Digestive Diseases 2017, 36, 1-14, doi:10.1159/000480149.
38. Wang, A.; Wakelee, H.A.; Aragaki, A.K.; Tang, J.Y.; Kurian, A.W.; Manson, J.E.; Stefanick, M.L. Protective Effects of Statins in Cancer: Should They Be Prescribed for High-Risk Patients? Current Atherosclerosis Reports 2016, 18, doi:10.1007/s11883-016-0625-y.
39. Tran-Duy, A.; Spaetgens, B.; Hoes, A.W.; de Wit, N.J.; Stehouwer, C.D.A. Use of Proton Pump Inhibitors and Risks of Fundic Gland Polyps and Gastric Cancer: Systematic Review and Meta-analysis. Clinical Gastroenterology and Hepatology 2016, 14, 1706-1719, doi:10.1016/j.cgh.2016.05.018.
40. Kong, P.; Wu, R.; Liu, X.; Liu, J.; Chen, S.; Ye, M.; Yang, C.; Song, Z.; He, W.; Yin, C.; et al. The Effects of Anti-inflammatory Drug Treatment in Gastric Cancer Prevention: an Update of a Meta-analysis. Journal of Cancer 2016, 7, 2247-2257, doi:10.7150/jca.16524.
41. Wright, E.; Schofield, P.T.; Molokhia, M. Bisphosphonates and evidence for association with esophageal and gastric cancer: A systematic review and metaanalysis. BMJ Open 2015, 5, doi:10.1136/bmjopen-2014-007133.
42. Stryjkowska-Gora, A.; Karczmarek-Borowska, B.; Gora, T.; Krawczak, K. Statins and cancers. Contemporary oncology (Poznan, Poland) 2015, 19, 167-175, doi:10.5114/wo.2014.44294.
43. Stegeman, I.; Bossuyt, P.M.; Yu, T.; Boyd, C.; Puhan, M.A. Aspirin for primary prevention of cardiovascular disease and cancer. A benefit and harm analysis. PLoS ONE 2015, 10, doi:10.1371/journal.pone.0127194.
44. Huang, W.-K.; Tu, H.-T.; See, L.-C. Aspirin Use on Incidence and Mortality of Gastrointestinal Cancers: Current State of Epidemiological Evidence. Current Pharmaceutical Design 2015, 21, 5108-5115, doi:10.2174/1381612821666150915110450.
45. Vallianou, N.G.; Kostantinou, A.; Kougias, M.; Kazazis, C. Statins and Cancer. Anti-Cancer Agents in Medicinal Chemistry 2014, 14, 706-712, doi:10.2174/1871520613666131129105035.
46. Song, H.; Zhu, J.; Lu, D. Long-term proton pump inhibitor (PPI) use and the development of gastric pre-malignant lesions. Cochrane Database of Systematic Reviews 2014, doi:10.1002/14651858.CD010623.pub2.
47. Ye, X.; Fu, J.; Yang, Y.; Gao, Y.; Liu, L.; Chen, S. Frequency-Risk and Duration-Risk Relationships between Aspirin Use and Gastric Cancer: A Systematic Review and Meta-Analysis. Plos One 2013, 8, doi:10.1371/journal.pone.0071522.
48. Wu, X.-D.; Zeng, K.; Xue, F.-Q.; Chen, J.-H.; Chen, Y.-Q. Statins are associated with reduced risk of gastric cancer: A meta-analysis. European Journal of Clinical Pharmacology 2013, 69, 1855-1860, doi:10.1007/s00228-013-1547-z.
49. Singh, P.P.; Singh, S. Statins are associated with reduced risk of gastric cancer: a systematic review and meta-analysis. Annals of oncology : official journal of the European Society for Medical Oncology 2013, 24, 1721-1730, doi:10.1093/annonc/mdt150.
50. Malek, M.; Aghili, R.; Emami, Z.; Khamseh, M.E. Risk of cancer in diabetes: The effect of metformin. ISRN Endocrinology 2013, 1, doi:10.1155/2013/636927.
51. Franciosi, M.; Lucisano, G.; Lapice, E.; Strippoli, G.F.M.; Pellegrini, F.; Nicolucci, A. Metformin Therapy and Risk of Cancer in Patients with Type 2 Diabetes: Systematic Review. Plos One 2013, 8, doi:10.1371/journal.pone.0071583.
52. Eslami, L.; Nasseri-Moghaddam, S. Meta-analyses: Does Long-term PPI use Increase the Risk of Gastric Premalignant Lesions? Archives of Iranian Medicine 2013, 16, 449-458.
53. Oh, Y.H.; Yoon, C.; Park, S.M. Bisphosphonate use and gastrointestinal tract cancer risk: Meta-analysis of observational studies. World Journal of Gastroenterology 2012, 18, 5779-5788, doi:10.3748/wjg.v18.i40.5779.

54. Colmers, I.N.; Bowker, S.L.; Johnson, J.A. Thiazolidinedione use and cancer incidence in type 2 diabetes: A systematic review and meta-analysis. Diabetes and Metabolism 2012, 38, 475-484, doi:10.1016/j.diabet.2012.06.003.
55. Bosetti, C.; Rosato, V.; Gallus, S.; Cuzick, J.; La Vecchia, C. Aspirin and cancer risk: a quantitative review to 2011. Annals of Oncology 2012, 23, 1403-1415, doi:10.1093/annonc/mds113.
56. Algra, A.M.; Rothwell, P.M. Effects of regular aspirin on long-term cancer incidence and metastasis: a systematic comparison of evidence from observational studies versus randomised trials. Lancet Oncology 2012, 13, 518-527, doi:10.1016/s1470-2045(12)70112-2.
57. Rothwell, P.M.; Fowkes, F.G.; Belch, J.F.; Ogawa, H.; Warlow, C.P.; Meade, T.W. Effect of daily aspirin on long-term risk of death due to cancer: analysis of individual patient data from randomised trials. Lancet 2011, 377, 31-41, doi:10.1016/s0140-6736(10)62110-1.
58. Yang, P.; Zhou, Y.; Chen, B.; Wan, H.-W.; Jia, G.-Q.; Bai, H.-L.; Wu, X.-T. Aspirin Use and the Risk of Gastric Cancer: A Meta-Analysis. Digestive Diseases and Sciences 2010, 55, 1533-1539, doi:10.1007/s10620-009-0915-0.
59. Matsushita, Y.; Sugihara, M.; Kaburagi, J.; Ozawa, M.; Iwashita, M.; Yoshida, S.; Saito, H.; Hattori, Y. Pravastatin use and cancer risk: A meta-analysis of individual patient data from long-term prospective controlled trials in Japan. Pharmacoepidemiology and Drug Safety 2010, 19, 196-202, doi:10.1002/pds.1870.
60. Kuoppala, J.; Lamminpaa, A.; Pukkala, E. Statins and cancer: A systematic review and meta-analysis. European Journal of Cancer 2008, 44, 2122-2132, doi:10.1016/j.ejca.2008.06.025.
61. Browning, D.R.; Martin, R.M. Statins and risk of cancer: a systematic review and metaanalysis. Int J Cancer 2007, 120, 833-843, doi:10.1002/ijc.22366.
62. Wang, W.H.; Huang, J.Q.; Zheng, G.F.; Lam, S.K.; Karlberg, J.; Wong, B.C.Y. Non-steroidal anti-inflammatory drug use and the risk of gastric cancer: A systematic review and meta-analysis. Jnci-Journal of the National Cancer Institute 2003, 95, 1784-1791, doi:10.1093/jnci/djg106.
63. Gonzalez-Perez, A.; Rodriguez, L.A.G.; Lopez-Ridaura, R. Effects of non-steroidal anti-inflammatory drugs on cancer sites other than the colon and rectum: a meta-analysis. Bmc Cancer 2003, 3, doi:10.1186/1471-2407-3-28.
64. Jolly, K.; Cheng, K.K.; Langman, M.J.S. NSAIDs and gastrointestinal cancer prevention. Drugs 2002, 62, 945-956, doi:10.2165/00003495-200262060-00006.
65. Bosetti, C.; Gallus, S.; La Vecchia, C. Aspirin and cancer risk: an update to 2001. European Journal of Cancer Prevention 2002, 11, 535-542, doi:10.1097/00008469-200212000-00005.
66. Abnet, C.C.; Freedman, N.D.; Kamangar, F.; Leitzmann, M.F.; Hollenbeck, A.R.; Schatzkin, A. Non-steroidal anti-inflammatory drugs and risk of gastric and oesophageal adenocarcinomas: results from a cohort study and a meta-analysis. British Journal of Cancer 2009, 100, 551-557, doi:10.1038/sj.bjc.6604880.
67. Ahn, J.S.; Eom, C.-S.; Jeon, C.Y.; Park, S.M. Acid suppressive drugs and gastric cancer: A meta-analysis of observational studies. World Journal of Gastroenterology 2013, 19, 2560-2568, doi:10.3748/wjg.v19.i16.2560.
68. Cai, D.; Qin, J.; Chen, G.; Feng, W.; Liu, J. Bisphosphonates use and risk of gastric cancer: an updated meta-analysis of cohort and case-control studies. Minerva Medica 2017, 108, 464-472, doi:10.23736/s0026-4806.17.05055-8.
69. Deng, Y.; Zhang, Z.; Jia, X.; Cheng, W.; Zhou, X.; Liu, Y.; Wang, M. Oral bisphosphonates and incidence of cancers in patients with osteoporosis: a systematic review and meta-analysis. Archives of Osteoporosis 2018, 14, doi:10.1007/s11657-018-0552-3.
70. Huang, X.-z.; Chen, Y.; Wu, J.; Zhang, X.; Wu, C.-c.; Zhang, C.-y.; Sun, S.-s.; Chen, W.-j. Aspirin and non-steroidal anti-inflammatory drugs use reduce gastric cancer risk: A dose-response meta-analysis. Oncotarget 2017, 8, 4781-4795, doi:10.18632/oncotarget.13591.
71. Ma, Z.; Wang, W.; Jin, G.; Chu, P.; Li, H. Effect of statins on gastric cancer incidence: A meta-Analysis of case control studies. Journal of Cancer Research and Therapeutics 2014, 10, 859-865, doi:10.4103/0973-1482.138218.
72. Tian, W.; Zhao, Y.; Liu, S.; Li, X. Meta-analysis on the relationship between nonsteroidal anti-inflammatory drug use and gastric cancer. European Journal of Cancer Prevention 2010, 19, 288-298, doi:10.1097/CEJ.0b013e328339648c.
73. Wang, L.; Zhang, R.; Yu, L.; Xiao, J.; Zhou, X.; Li, X.; Song, P.; Li, X. Aspirin Use and Common Cancer Risk: A Meta-Analysis of Cohort Studies and Randomized Controlled Trials. Frontiers in Oncology 2021, 11, doi:10.3389/fonc.2021.690219.

74. You, S.; Sun, G.; Yao, Q.; Wan, Z.; Huang, X. Statin use and risk of gastrointestinal cancer: a meta-analysis of cohort studies. International Journal of Clinical and Experimental Medicine 2018, 11, 1437-1447.
75. Zeng, R.; Cheng, Y.; Luo, D.; Wang, J.; Yang, J.; Jiang, L.; Zhuo, Z.; Guo, K.; Wu, H.; Leung, F.W.; et al. Comprehensive analysis of proton pump inhibitors and risk of digestive tract cancers. European Journal of Cancer 2021, 156, 190-201, doi:10.1016/j.ejca.2021.07.030.
76. Zhang, K.; Bai, P.; Dai, H.; Deng, Z. Metformin and risk of cancer among patients with type 2 diabetes mellitus: A systematic review and meta-analysis. Primary Care Diabetes 2021, 15, 52-58, doi:10.1016/j.pcd.2020.06.001.
